# Damage Evolution Characteristics of Anti-Slide Piles in Loess Landslides and a Possible Characterization Method

**DOI:** 10.3390/s26010192

**Published:** 2025-12-27

**Authors:** Tong Zhao, Wei Yang, Suya Zheng, Xunchang Li, Zheng Lu

**Affiliations:** 1 College of Geological Engineering and Geomatics, Chang’an University, Xi’an 710054, China; 2 State Key Laboratory of Loess Science, Xi’an 710054, China; 3 Key Laboratory of Western China’s Mineral Resources and Geological Engineering, Ministry of Education, Xi’an 710054, China; 4 Key Laboratory of Ecological Geology and Disaster Prevention, Ministry of Natural Resources, Xi’an 710054, China; 5 Northwest Electric Power Design Institute Co., Ltd. of China Power Engineering Consulting Group, Xi’an 710054, China

**Keywords:** acoustic emission, anti-slide pile failure, monitoring technology

## Abstract

Effective monitoring and early warning of the instability of anti-slide piles in loess landslides depend on identifying the precursory signs of anti-slide pile failure. The acoustic emission (AE) characteristics of concrete anti-slide piles under cyclic loading were studied by using the model box test of the loess landslide–pile system. Cyclic graded loading simulates natural landslide sliding. The synergistic relationship between AE signal characteristics and pile bending moment is established, which reveals the evolution law from micro-damage to macro-damage. The results show that (1) AE ringing count and energy count change in the same way, first stable and then a sudden increase. The evolution of AE dominant frequency and amplitude experiences four stages: low frequency and low amplitude (initial damage), high frequency and low amplitude (stable development), medium frequency and high amplitude (accelerated development), and low frequency and high amplitude (failure). Each stage obviously corresponds to the change in bending moment. (3) The significant increase in the proportion of low-frequency AE energy effectively indicates that the landslide–pile system has entered the state of accelerated deformation and instability, which provides a quantifiable, real-time early warning criterion. This study verifies the feasibility and effectiveness of acoustic emission technology in anti-slide pile damage monitoring and landslide early warning and provides a new technical way for the precursor’s identification and early warning of anti-slide pile instability.

## 1. Introduction

The Loess Plateau of China features a distinctive geographical and geological setting, characterized by frequent and widespread geological hazards [1]. Loess, with its high porosity and well-developed vertical joints, exhibits an under-consolidated structure and pronounced collapsibility [2,3,4]. When water from rainfall or irrigation infiltrates along preferential flow paths, the shear strength of the slope soil deteriorates rapidly, often triggering landslides. As a typical and highly destructive type of geological disaster, loess landslides are widely distributed, occur frequently, have complex triggering mechanisms, and cause severe damage. Under the combined pressures of natural environmental variability and increasing human engineering activities, loess landslide disasters are becoming more frequent, posing serious threats to local lives and property, the safe operation of major infrastructure, and sustainable socio-economic development. Research on effective landslide mitigation is therefore both urgent and critically important, forming a key component of regional disaster prevention and reduction efforts [5,6,7,8].

Anti-slide piles are widely used in the treatment of loess landslides due to their advantages such as flexible embedment, straightforward construction, minimal excavation requirements, and effective resistance to sliding [9,10]. By installing anti-slide piles, landslide deformation can be effectively constrained, thereby reducing associated hazards [11]. However, the support performance of anti-slide piles may degrade under various influencing factors, eventually leading to failure in restraining landslide movement and compromising slope stability. Influenced by extreme rainfall and human engineering activities, landslides continue to threaten road infrastructure and passing vehicles. Therefore, monitoring and early warning of anti-slide piles are of significant importance [12,13]. Conducting damage detection on anti-slide piles and implementing timely countermeasures can help prevent potential problems and enhance overall landslide risk management.

Currently, the condition monitoring of anti-slide piles primarily relies on measuring three key parameters: displacement, material stress, and external loads. Pile top displacement is typically observed periodically using a total station, while lateral displacement is continuously monitored by installing fixed inclinometers within boreholes. For stress monitoring, internal concrete stress is measured using embedded vibrating wire or fiber-optic strain gauges; however, these methods are hindered by complex installation procedures, low survival rates, and interference with subsequent concrete placement. Reinforcing bar stress is commonly monitored using welded strain gauges, which, although mature and relatively simple to install, lack sufficient sensitivity to detect small strains and often fail to capture early micro-deformation signals associated with initial damage. Landslide thrust is usually quantified using earth pressure cells installed in the soil behind the pile; however, readings are affected by contact conditions, stress concentrations, and long-term creep, compromising data reliability over time.

Existing monitoring techniques are predominantly discrete point-based, with limited sensor coverage that results in low spatial resolution, poor data representativeness, and labor-intensive data processing. More critically, these approaches are indirect: they infer internal damage—such as crack initiation, propagation, and rebar yielding—by analyzing observed “performance parameters” (displacement, strain, and earth pressure) in conjunction with theoretical models. Such indirect diagnostics are prone to ambiguity, lag, and they cannot visually or continuously reveal the actual failure mode or extent of damage within the buried pile. When anomalies occur, engineers often resort to costly and disruptive excavation to verify the pile’s true condition. This practice underscores the limitations of current monitoring systems and highlights the urgent need for more direct, spatially continuous, and real-time assessment methods.

With the continuous advancement of monitoring technologies, more sophisticated methods have been implemented for the health monitoring of anti-slide piles. Fiber-optic sensing technology, which uses light as the signal carrier and optical fibers as the transmission medium, integrates sensing and data transmission into a single system. It offers significant advantages, such as real-time response, high precision, fully distributed measurement, long-distance capability, and strong resistance to electromagnetic interference [14,15,16,17,18,19]. Consequently, it has become a prominent focus in current research and applications for structural deformation monitoring. In recent years, distributed fiber-optic sensing (DFOS) technology has been widely adopted in geohazard monitoring. Its key features—including immunity to electromagnetic interference, truly distributed sensing along the fiber, and the ability to monitor multiple physical parameters—have enabled its application in deformation monitoring of various types of pile foundations, providing a more continuous and spatially detailed understanding of structural behavior [20].

However, existing monitoring techniques still lack effective methods to capture the instantaneous and continuous evolution of internal damage in concrete anti-slide piles. As a quasi-brittle material, concrete undergoes progressive fracturing: when cracking initiates, failure is not instantaneous; instead, a fracture process zone gradually develops ahead of the crack tip, eventually leading to a loss of load-bearing capacity. The core of this failure process lies in the initiation and propagation of internal microcracks. This damage evolution is accompanied by the release of strain energy in the form of elastic waves—precisely, the signals detected by acoustic emission (AE) technology [21]. By directly capturing these internal fracture signals, AE enables the characterization of micro-damage mechanisms throughout the deformation process, revealing the complete sequence, from microcrack initiation and growth to final coalescence into macroscopic fractures.

AE technology employs a passive monitoring method that enables real-time, dynamic tracking of the deformation and failure processes of anti-slip piles without requiring external signal sources. By detecting acoustic signals generated within the material, acoustic emission technology accurately reflects changes in the internal state and the development of defects, allowing continuous and dynamic monitoring of the damage progression. Due to its high sensitivity, this technology can fully capture the entire crack evolution process, from initiation to propagation, and is not limited by the type of material or structure [22,23,24]. Leveraging these significant advantages, acoustic emission technology shows great potential for non-destructive testing of concrete [25]. The real-time data obtained through this technology allow precise detection of damage signals within concrete anti-slip piles, facilitate in-depth analysis of the damage mechanisms, and systematically reveal the overall failure modes of the components.

AE technology has been extensively applied in concrete-related research due to its ability to capture the evolution of internal damage. Numerous studies have demonstrated its effectiveness in various contexts. For example, Wei et al. showed that AE data enable accurate localization of damage sources and crack mapping during crack propagation [26]. Chen et al., through three-point bending tests, identified distinct turning points in cumulative AE hits and ring-down counts, correlating AE event rates with concrete crack width [27]. Xie et al. used AE to delineate the failure stages of porous concrete under uniaxial compression into initial, crack propagation, and failure phases [28]. Hua et al. investigated how fiber content and loading rate influence the mechanical and damage behavior of basalt fiber-reinforced concrete via AE monitoring [29]. Ren et al. linked AE parameters to internal damage in steel–fiber concrete, noting signal variations across failure stages [30]. Wu et al. associated AE signal features with concrete fracture stages, suggesting that high-frequency energy decay serves as an early warning indicator [31]. Chen et al. applied AE to analyze fracture energy evolution in steel–slag porous concrete, confirming correlations between fracture energy and AE energy release [32].

Collectively, these studies confirm that AE technology can effectively characterize the entire damage evolution process—from microcrack initiation and growth to macroscopic fracture—by detecting elastic waves released during concrete damage. This provides a reliable method for damage identification and fracture warning in concrete structures and offers a theoretical foundation for analyzing damage evolution in anti-slide piles using AE, as undertaken in the present study.

The application of acoustic emission technology for monitoring damage in anti-slide piles presents a series of unique challenges due to the complexity of landslide mechanisms. These challenges stem from fundamental differences in the stress conditions experienced by anti-slide piles in real landslide environments compared to the behavior of conventional concrete materials under standard laboratory test conditions [33]. The landslide process is dynamic, multi-staged, and non-uniform, causing anti-slide piles to be subjected to complex, time- and space-varying composite loads. Unlike standard concrete specimens tested under unidirectional or simple cyclic loads in the laboratory, anti-slide piles under landslide thrust simultaneously endure intense coupling effects of shear, bending, and axial forces, resulting in complex and highly nonlinear stress paths. This intricate stress state complicates the correlation between classical AE parameters (such as ringing count, energy release, and b-value), which are derived from simplified loading modes, and the actual damage evolution of the pile. Furthermore, current damage warning thresholds based on AE data predominantly originate from statistical analyses of standard laboratory specimens, failing to fully account for the dynamic coupling relationships among different stages of landslide evolution—such as initial deformation, uniform sliding, and accelerated failure—and the corresponding damage states of anti-slide piles.

The instability mechanisms of landslides are highly complex and uncertain, which limits the practical applicability of traditional analysis methods based on deterministic models [34]. Moreover, under dynamic landslide thrust, the loading conditions of anti-slide piles differ fundamentally from those of laboratory specimens subjected to static or simple cyclic loads. In reality, piles experience complex coupled actions involving varying lateral forces, bending moments, and shear stresses, resulting in stress paths far more intricate than those observed in standard concrete specimens. This study focuses on the characteristic behavior of “push-type landslides”, in which the sliding mass often undergoes an intermittent cyclic process of “sliding → equilibrium → renewed sliding”, rather than following a simple progressive failure path. Macroscopically, this behavior manifests as a dynamic quasi-stable state. However, once external triggers—such as intense rainfall, seismic activity, or human disturbances—exceed the stability threshold of a given phase, the quasi-equilibrium is disrupted, and the slope re-enters an active sliding stage. This cycle repeats until accumulated deformation ultimately leads to complete slope failure.

To investigate the complete damage-to-failure process of an anti-slide pile subjected to intermittent and dynamically varying landslide thrust, this study employs a cyclic progressive loading test. Using an indoor physical model, this approach actively replicates the landslide’s progressive failure cycle of “sliding → equilibrium → sliding again → equilibrium again”, thereby simulating the intermittent application and release of landslide thrust. By recreating the complex stress history experienced by anti-slide piles experience in service, the test provides a reliable experimental basis for elucidating the intrinsic relationship between pile damage evolution and its AE response.

## 2. Test on the Deformation and Failure of Anti-Sliding Piles

Landslides can be classified into two primary types based on distinct mechanical mechanisms: traction-type and push-type landslides. A traction-type landslide occurs when the lower section begins to slide, causing a loss of support for the upper section, which then deforms and slides. In contrast, a push-type landslide is characterized by the upper rock layer sliding and compressing the lower section, resulting in deformation. Notably, push-type landslides exhibit higher sliding velocities, and their sliding surfaces typically feature wavy undulations. These landslides predominantly occur on slopes composed of accumulated materials.

This study investigates the damage evolution process of push-type loess landslides during their sliding phase. Such landslides typically experience a prolonged creep stage, progressing from initial deformation to eventual instability. The deformation of the landslide body exhibits dynamic stability, meaning the deformation rate may temporarily stabilize over a certain period. However, when subjected to new external disturbances—such as rainfall, earthquakes, or human activities—the equilibrium state is disrupted, causing the landslide body to re-enter an active deformation and sliding phase. This cyclic process continues until the landslide body loses stability entirely, ultimately triggering failure. By employing cyclic loading, this study effectively simulates the complex evolutionary pattern of “sliding → equilibrium → sliding → re-equilibrium”, providing critical experimental insights into the dynamic mechanisms of landslides and their mitigation strategies. The load increment at each stage is 4 kPa, the holding time for each stage load is 1800 s, and the cyclic loading frequency is 0.5 Hz.

Similarity theory defines the conditions under which physical phenomena can be considered analogous and describes the governing laws of such systems. As the theoretical foundation for physical modeling, it provides essential similarity criteria that enable the design of scaled experiments. By applying this theory, prototype structures can be represented by indoor physical models that meet all required similarity conditions, thereby offering reliable guidance for model design and specimen fabrication. The theory is organized around three fundamental theorems, which systematically address the properties of similarity, dimensional systems, and the necessary conditions for achieving and maintaining similitude.

The physical model tests were conducted in a custom-designed test box with a geometric similarity ratio (CL) of 20. Specific similarities are shown in Table 1. The internal dimensions of the test box were 4.3 m (length) × 2.5 m (width) × 3 m (height) The rigid frame structure was constructed using welded square steel and channel steel, while the sidewalls were fitted with 10-mm-thick tempered glass panels to facilitate visual monitoring of soil deformation. A hydraulically adjustable crossbeam was installed at the top of the test box to apply controlled loading conditions.

To meet the required similarity ratio, the similarity of the anti-slide piles is discussed below [35].

Applying the second similarity theorem to the elastic mechanics model yields the following parameter expressions:

(1) $$f\left(E,\sigma ,\bar{\sigma },\varepsilon ,\delta ,\mu ,x,l\right)=0.$$

By dimensionalizing it, we can obtain

(2) $$E=F\cdot L^{-2},\sigma =F\cdot L^{-2},\bar{\sigma }=F\cdot L^{-2},\varepsilon =1,\delta =L,\mu =1,x=F\cdot L^{-3},l=L.$$

Six π terms can be obtained from Formula (1); then, there is

(3) $$f^{\prime }(\pi_{1},\pi_{2},\pi_{3},\pi_{4},\pi_{5},\pi_{6})=0.$$

The formula can represent the same physical phenomenon as *f* = 0. By taking the volume force *x* and the length (*l*) as the fundamental dimensions—where volume force has dimensions *FL*^−3^ and length has dimension *l*—and then expressing each π term in terms of these fundamental dimensions, we obtain

(4) $$\pi_{1}=\frac{E}{\chi^{\alpha }p^{\beta }}=\frac{FL^{-2}}{{\left(FL^{-3}\right)}^{\alpha }L^{\beta }}=\frac{F^{1-\alpha }}{L^{2-3\alpha +\beta }}.$$

When *α* = 1 and *β* = 1, π_1_ becomes a dimensionless quantity, as shown below:

(5) $$\pi_{1}=\frac{E}{x^{l}}.$$

Similarly, we can get

(6) $$\pi_{2}=\frac{\sigma }{xl};\pi_{3}=\frac{\bar{\sigma }}{xl};\pi_{4}=\varepsilon ;\pi_{5}=\frac{\delta }{l};\pi_{6}=\mu .$$

According to the similarity criterion of mechanical phenomena,

(7) $$\frac{C_{\sigma }}{C_{x}C_{\iota }}=1,\frac{C_{E}}{C_{x}C_{\iota }}=1,\frac{C_{\bar{\sigma }}}{C_{x}C_{\iota }}=1,C_{\varepsilon }=1;C_{\mu }=1.$$

Bringing $C_{l}$ $=20\;and\;C_{\sigma }=20$ into Formula (5), there are

(8) $$C_{x}=1,C_{E}=20,C_{\bar{\sigma }}=20,C_{\varepsilon }=1,C_{\mu }=1.$$

Reinforcement Calculation of the Model Pile in the Model Test:

(9) $$C_{m}=\frac{M_{m}}{M_{p}}=\frac{f_{mp}A_{m}}{f_{pp}A_{p}}h_{m0}=\frac{A_{m}}{A_{p}}C_{D}C_{L}.$$

Reinforcement Area:

(10) $$A_{ms}=\frac{A_{ps}f_{py}}{A_{pc}f_{pc}}\cdot \frac{A_{mc}f_{mc}}{f_{my}}=\frac{1}{C_{L}^{2}}\cdot \frac{f_{pc}}{f_{pc}}\cdot \frac{f_{py}}{f_{my}}\cdot A_{ps}.$$

Shear Similarity Constant:

(11) $$C_{V}=\frac{V_{m}}{V_{p}}=\frac{f_{my}A_{ms}}{f_{py}A_{ps}}=\frac{A_{ms}}{A_{ps}}C_{v}.$$

The area of stirrup reinforcement:

(12) $$A_{svm}=\frac{A_{ps\nu }f_{pyv}h_{p0}}{f_{my\nu }h_{m0}}C_{\nu }$$

In the formula:

*A_ps_*—the reinforcement area of the prototype.

*A_pc_*—the design value of the compressive strength of the prototype structure concrete.

*F_py_*—the tensile strength design value of the stressed steel bar of the prototype structure.

*A_ms_*—the reinforcement area of the model structure.

*A_mc_*—the gypsum area of the model structure.

*f_mc_*—the design value of the gypsum compressive strength of gypsum in the model structure.

*f_my_*—the design value of the tensile strength of the aluminum rod in the model structure.

In the test, when *f_y_ = f_y_^′^ = f_my_ =* 100 N/mm^2^, *M =* 607.61 N m, *b =* 50 mm, *h*_0_ = 69 mm, *α*_1_ = 1.0, *ξ_b_ =* 0.6, and *f_c_ = f_mc_ =* 7.3 N/mm^2^.

**Figure 1:**
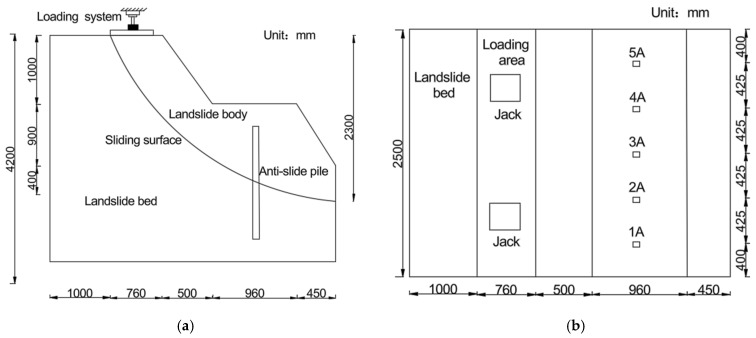
Model schematic. (**a**) Model elevation schematic; (**b**) model plan schematic.

The sliding bed material used in this experiment consisted of remolded loess. Nine fully embedded concrete anti-slide piles, each 90 cm in length, were installed, with material properties equivalent to C10-grade concrete. Each pile featured an embedded section and a stressed section of equal length (45 cm). Strain gauges were mounted on the reinforcing bars within the pile body to monitor strain distribution. The piles were arranged with a center-to-center spacing of 42.5 cm, and their cross-sectional dimensions were 80 mm (width) × 50 mm (height). The experimental setup is illustrated in Figure 1.

In the model test, the spacing of the anti-slip piles was determined using a 1:20 geometric similarity ratio. The prototype pile spacing calculated from this conversion was 8.5 m, which falls within the recommended range of 5–10 m specified in the Code for the Design of Landslide Stabilization [36]. For safety reasons, conservative values (e.g., 5 m) are often adopted in actual engineering projects. As an exploratory study, this research focuses on elucidating the response mechanism between pile damage evolution and acoustic emission signals under landslide thrust. To effectively capture pile failure signals and emphasize landslide–pile interactions, a larger pile spacing (8.5 m) within the code’s allowances was selected to facilitate experimental observation and signal identification.

The experimental setup utilized homogeneous loess for both the sliding bed and sliding mass. The loess was first saturated to achieve its optimum moisture content and then compacted in layers following a standardized procedure. Each uncompacted layer had a thickness of 200 mm, which was reduced to 150 mm after compaction. A bilayer polyethylene film was installed to simulate the sliding zone, featuring an artificially created arcuate failure surface. The internal friction angle of the double-layer plastic film typically ranges from 10° to 20°. Based on the load capacity at which the landslide mass reaches ultimate equilibrium without the installation of anti-slip piles [37], the internal friction angle of the slip surface was calculated to be 16°, consistent with actual conditions. The geotechnical properties of the test soil, determined through standard laboratory tests, are summarized in Table 2.

**Figure 2:**
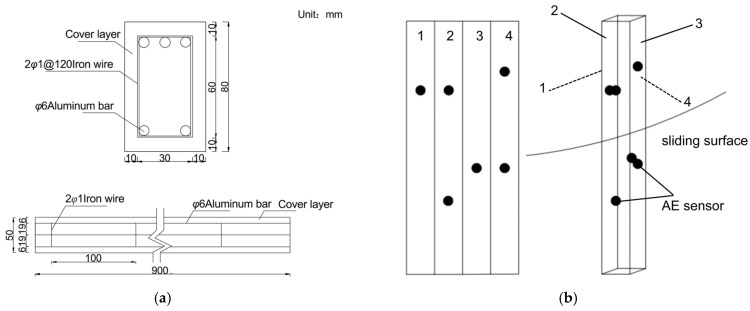
Introduction to the anti-slip piles. (**a**) Anti-slide pile structure; (**b**) installation position of the AE sensors.

AE signals were acquired using piezoelectric AE sensors. Six AE sensor probes were strategically mounted on the anti-slide pile shaft to ensure accurate monitoring of damage-induced AE signals during the pile failure process. The sensor configuration is illustrated in Figure 2.

The isotropic nature of aluminum ensures uniform signal propagation in all directions, while its exceptional electrical and thermal conductivity enhances its reliability in diverse industrial applications. Consequently, aluminum rods were employed to simulate steel reinforcement used in the pile body.

Before installing the sensor, the surface at the pile measurement points must be ground and cleaned to ensure the sensor adheres flush and securely to the pile surface. Next, apply a layer of petroleum jelly to the pile surface to lubricate and reduce friction between the sensor and the test surface. Then, firmly press the acoustic emission sensor against the designated measurement point and secure it with adhesive tape to prevent displacement or detachment during loading.

This test utilized the G80 narrow-band AE sensor manufactured by QAWRUMS (Guangzhou, China). Before starting, the system was calibrated using a standard lead break test, which included verification of sensor and channel functionality, wave velocity calibration, positioning accuracy assessment, and system sensitivity validation. The system parameters were configured as follows: the threshold was set to 10 mV, preamplifier gain at 40 dB, filter in passband mode, and sampling frequency at 769 kHz. Acoustic emission characteristic parameters were set as follows: peak definition time (PDT) at 150 μs, hit definition time (HDT) at 600 μs, and hit lockout time (HLT) at 1000 μs. The sensor has a resonant frequency of 80 kHz and an effective operating frequency range of 20–180 kHz.

The AE monitoring system used in this study was the SAEU3H model (Beijing Shenghua Xingye Technology Co., Ltd., Beijing, China), which employs USB 3.0 and utilizes communication for real-time data transmission to a host computer. The system enables direct storage of data during loading. Each SAEU3 AE acquisition card features four independent channels, each equipped with a 16-bit A/D converter supporting multiple sampling frequencies (1.5, 3, 5, 10, and 40 MHz) The maximum sampling rate per channel is 10 MS/s (megasamples per second), with continuously adjustable sampling rates, a dynamic range of 9 dB, and a maximum single-waveform sampling length of 128 k points per channel.

## 3. Analysis of Acoustic Emission Signals During the Failure Process of Anti-Slide Piles

### 3.1. Time Domain Analysis of Acoustic Emission Signals for Anti-Slide Piles Under Cyclic Load

#### 3.1.1. Ringing Count Distribution Characteristics

**Figure 3:**
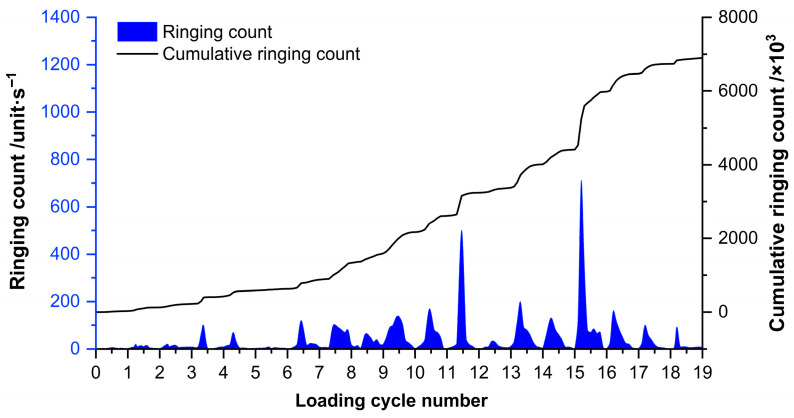
Time course curve of the ringing count of acoustic emission signals.

The AE ringing count rate serves as a reliable indicator of AE event frequency. Figure 3 illustrates the evolution of this characteristic AE parameter throughout the cyclic loading process of the concrete anti-slide pile, revealing distinct behavioral patterns.

The variation in the ring count of the AE signal can be divided into four stages. The first stage (Stage I) spans the first to sixth loading cycles, during which ring count fluctuations remain minimal, with only a few signals exhibiting increased intensity. The second stage (Stage II) covers the seventh to twelfth loading cycles and is characterized by pronounced ring count fluctuations, indicating that the anti-slip pile has entered the second stage of failure. The third stage (Stage III) occurs during the thirteenth to fifteenth loading cycles, where ring count changes are significant but not extreme. The fourth stage (Stage IV) begins with the sixteenth loading cycle and continues until completion, during which the ring count reaches its peak. Subsequently, even with increases in the upper limit load, changes in the ring count become gradual, indicating that the material has entered a new failure stage after the sixteenth loading cycle.

Analysis of the cumulative ringing count reveals a lower count rate during the early stages of cyclic loading, with a higher count rate observed during the mid-to-late stages. The ringing count exhibits abrupt changes during the twelfth and sixteenth loading cycles, indicating that significant alterations in the material’s internal state occur at these points.

#### 3.1.2. Energy Characteristics During Cyclic Loading

**Figure 4:**
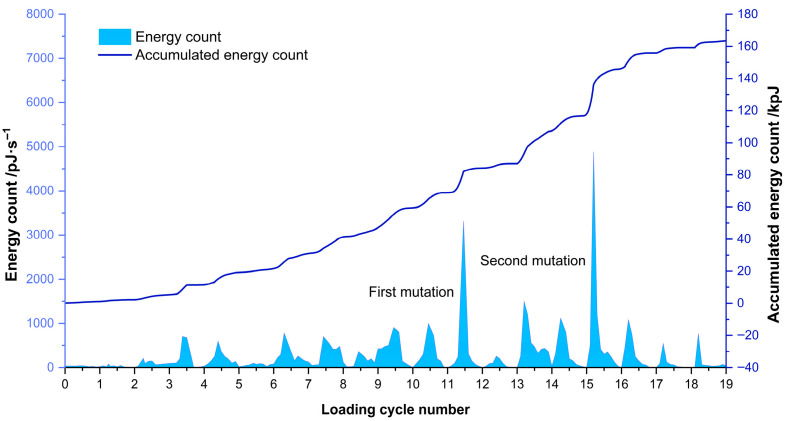
Energy of acoustic emission signals, and time history curves of the loads.

The AE energy count serves as a reliable indicator of signal intensity. As illustrated in Figure 4, the AE energy count displays distinct evolutionary patterns throughout the cyclic loading process of concrete anti-slide piles.

During the initial three loading cycles, the energy count remained relatively stable. However, a significant increase in energy release was observed during the fourth loading cycle, followed by a moderate decrease in the fifth cycle. The sixth cycle exhibited comparatively low signal activity.

From the seventh to the eleventh cycles, the system underwent a progressive phase of energy accumulation. This trend was abruptly interrupted during the twelfth cycle, which was characterized by dramatic fluctuations in energy counts. Subsequent cycles exhibited attenuated AE signals, indicating another period of energy accumulation. The sixteenth cycle displayed extreme signal intensity, accompanied by a sharp surge in elastic energy.

A detailed analysis of the AE energy evolution reveals distinct characteristics across different loading stages: initial signal stability during the early loading phases, followed by a moderate energy increase at the fourth cycle. Intermediate cycles exhibited substantial energy variation, while the twelfth cycle showed an abrupt energy transition. Complete energy release occurred at the sixteenth cycle, as evidenced by sudden changes in the ringing counts and a significant cumulative energy increase. Notably, the AE signal generation points exhibited temporal advancement, with prolific signal emission occurring prior to reaching previous peak loads.

### 3.2. Frequency Domain Analysis of Acoustic Emission Signals for Anti-Slip Piles Under Cyclic Load

Under cyclic loading, concrete anti-slide piles generate time-dependent AE signals characterized by stochastic behavior and non-stationary characteristics. These signals exhibit distinct frequency-phase distributions. To accurately capture their instantaneous features (e.g., instantaneous frequency and amplitude), Hilbert–Huang transform (HHT) proves particularly suitable. Unlike Fourier transform, which excels in analyzing stationary signals but fails to track temporal frequency variations effectively, HHT offers superior adaptability and high time–frequency resolution [38].

HHT represents an advanced signal processing technique that combines the empirical mode decomposition (EMD) method with Hilbert spectral analysis. Firstly, the EMD algorithm adaptively decomposes non-stationary signals into a finite set of intrinsic mode functions (IMFs), each representing distinct oscillatory modes embedded within the original signal. Then, Hilbert transform is applied to each IMF component to extract instantaneous frequency characteristics. This combined approach enables the construction of a high-resolution time–frequency representation, known as the Hilbert spectrum, which effectively captures the nonlinear and non-stationary features of acoustic emission signals [39].

The processed data were transformed into the Hilbert spectra to characterize the time–frequency probability distributions of the signals. This transformation enabled the identification of temporal evolution patterns in both signal energy and frequency components, facilitating subsequent in-depth analysis.

**Figure 5:**
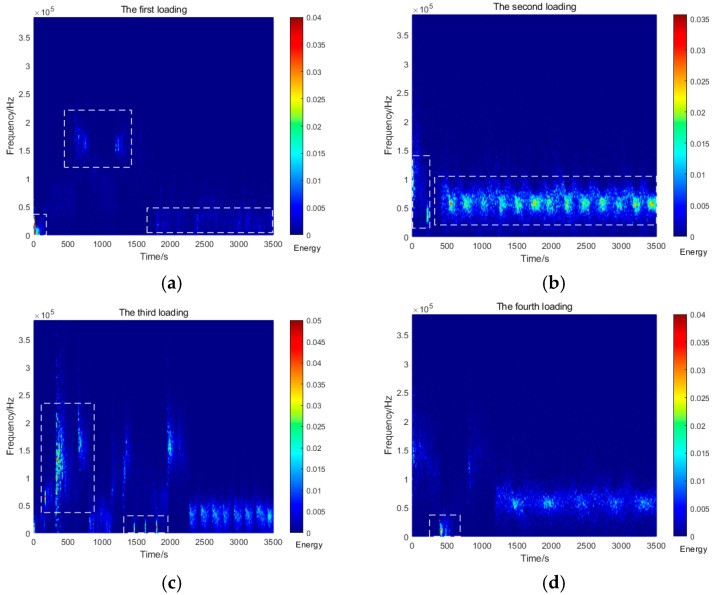
AE Spectrum graphs for the first to the fourth loadings. (**a**) First loading; (**b**) second loading; (**c**) third loading; (**d**) fourth loading.

**Figure 6:**
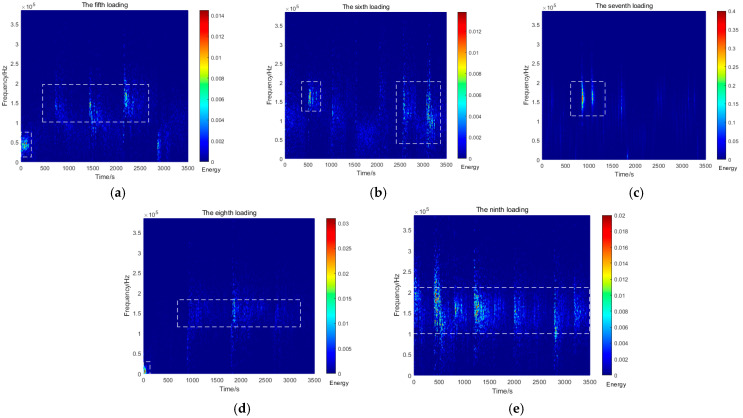
AE spectrum graphs for the fifth to the ninth loadings. (**a**) Fifth loading; (**b**) sixth loading; (**c**) seventh loading; (**d**) eighth loading; (**e**) ninth loading.

**Figure 7:**
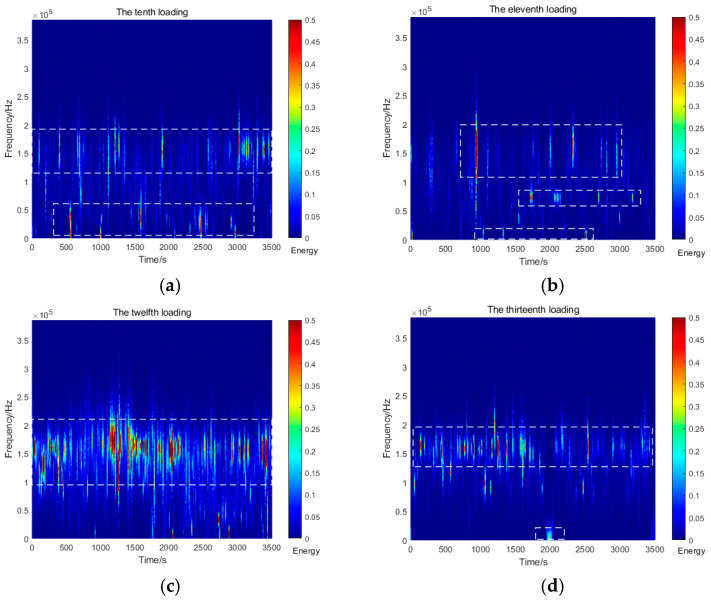
AE spectrum graphs for the tenth to the thirteenth loadings. (**a**) Tenth loading; (**b**) eleventh loading; (**c**) twelfth loading; (**d**) thirteenth loading.

**Figure 8:**
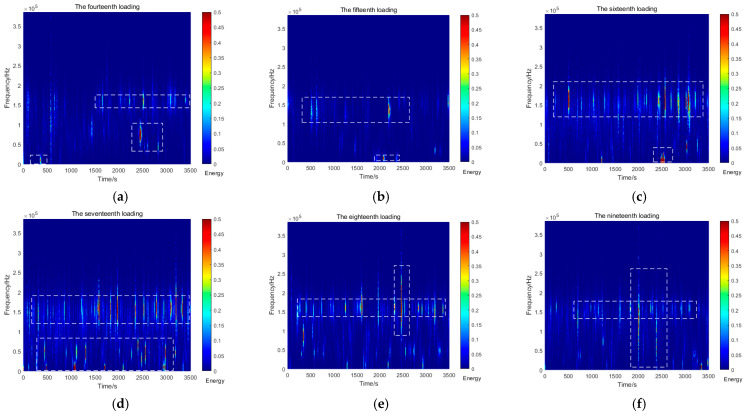
AE spectrum graphs from the fourteenth to the nineteenth loadings. (**a**) Fourteenth loading; (**b**) fifteenth loading; (**c**) sixteenth loading; (**d**) seventeenth loading; (**e**) eighteenth loading; (**f**) nineteenth loading.

Figure 5 illustrates the evolutionary characteristics of AE signals during Stage I (4, 8, 12, and 16 kPa). During the first loading (4 kPa), AE signals exhibited relatively low intensity, with dominant frequency components distributed within two distinct bands: 0–50 kHz and 150–200 kHz. As the load increased, significant variations emerged in the dominant frequency bands, while the energy evolution demonstrated greater stability, maintaining fluctuations below 0.1 throughout these early loading cycles. The physical quantity represented by the color bars in Figure 5, Figure 6, Figure 7 and Figure 8 is energy density [40].

Figure 6 illustrates the variations in AE signals during Stage II (20, 24, 28, 32, and 36 kPa). Throughout these loading cycles, the signal energy exhibited minimal fluctuations, remaining within the range of 0–0.4. Notably, the dominant frequency band progressively widened from an initial 0–200 kHz to 0–300 kHz. A significant amplitude increase (up to 0.4) was observed during the seventh loading cycle. Although the energy variation during the eighth loading cycle was relatively minor compared to the sixth, a substantial expansion of the main frequency band occurred at this stage.

This loading phase demonstrates two key characteristics: a continuous expansion of the main frequency band with the increasing load, accompanied by an abrupt energy surge reaching 20 times the baseline level, and significant alterations in both signal frequency and energy. Following these intense material responses, the system entered a stabilization phase primarily marked by elevated signal frequency and energy levels.

Figure 7 illustrates the variation characteristics of AE signals during Stage III (40, 44, 48, and 52 kPa). At this stage, the AE energy exhibits significant fluctuations. Specifically, the energy remains below 1.0 during the 10th loading cycle but stabilizes between 3.5 and 4.0 in subsequent cycles. The dominant AE frequencies are distributed in two distinct bands: 0–50 kHz (low-frequency) and 100–200 kHz (high-frequency). As the cyclic loading progresses, the amplitude and frequency of the AE signals increase with the applied load. Notably, the low-frequency signals become more pronounced, while the AE energy gradually rises in response to the increasing load.

Figure 8 illustrates the variations in AE signals recorded during Stage IV (56, 60, 64, 68, 72, and a second 68 kPa). A marked increase in low-frequency signals (0–50 kHz) was observed during the 15th and 16th loading cycles, with the low-frequency energy reaching approximately 16 units during the 16th cycle. In the 17th loading cycle, although the overall energy decreased, persistent high-energy signals remained detectable in the low-frequency range, coinciding with significant landslide displacement. The final two loading cycles were characterized predominantly by high-frequency signals with substantially reduced energy levels.

**Figure 9:**
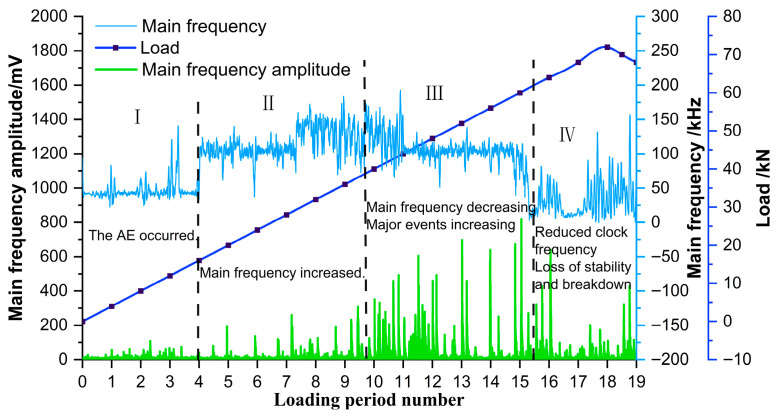
Main frequency–amplitude time history curve of the acoustic emission signal.

To comprehensively characterize the spectral properties of the AE signals, the dominant frequency and amplitude within each unit time interval were extracted, and their variations with the loading force are presented in Figure 9.

During the initial four loading cycles (Stage I: 0–16 kN), the AE signals exhibited a main frequency concentrated around 50 kHz, with amplitudes generally below 100 mV and only sporadic increases. The damage variable remained low, AE hits were limited, and the peak frequency distribution was mainly in the low-frequency range, indicating weak AE activity and low energy release. From the fifth to the eleventh loading cycles (Stage II: 16–44 kN), the main frequency shifted markedly with the increasing load: it migrated to 100 kHz between 16 and 28 kN, rose sharply to about 150 kHz at 28–36 kN, and then dropped back to 100 kHz during 36–44 kN. In this stage, the damage variable grew steadily, AE hits increased explosively—accounting for approximately 50% of the total—and signal amplitudes fluctuated strongly, reaching 400–600 mV in some instances, reflecting significantly enhanced AE activity and energy release. The twelfth to fifteenth loading cycles (Stage III: 44–60 kN) corresponded to the accelerated damage phase. The main frequency gradually fell below 100 kHz, and the frequency band narrowed. AE activity continued to rise, with signal peaks concentrated in the mid- and mid-high-frequency bands (the latter being especially dense). Amplitudes generally exceeded 300 mV, some lower-amplitude signals disappeared, and the damage variable increased rapidly toward a critical level, marking the transition of the pile into an unstable damage state. During the sixteenth to nineteenth loading cycles (Stage IV), failure occurred. The main frequency further decreased to 0–50 kHz, showing an overall trend of “rise–peak–sharp decline”. The damage variable stabilized and signal amplitudes declined noticeably, varying only slightly under continued loading.

In summary, the evolution of the dominant frequency and amplitude of AE signals from the anti-slide pile follows four distinct stages: low frequency and low amplitude (Stage I) → high frequency and low amplitude (Stage II) → mid frequency and high amplitude (Stage III) → low frequency and high amplitude (Stage IV).

## 4. Discussion

### 4.1. Analysis of the Characteristics of the AE RA Values and Average Frequency AF Values

RA–AF correlation analysis is a method used to identify fracture types and damage progression in material components. RA (rise time/amplitude ratio) is defined as the ratio of rise time to signal amplitude, expressed in ms/V. AF (average frequency) refers to the ratio of ringing count to signal duration, measured in kHz.

**Figure 10:**
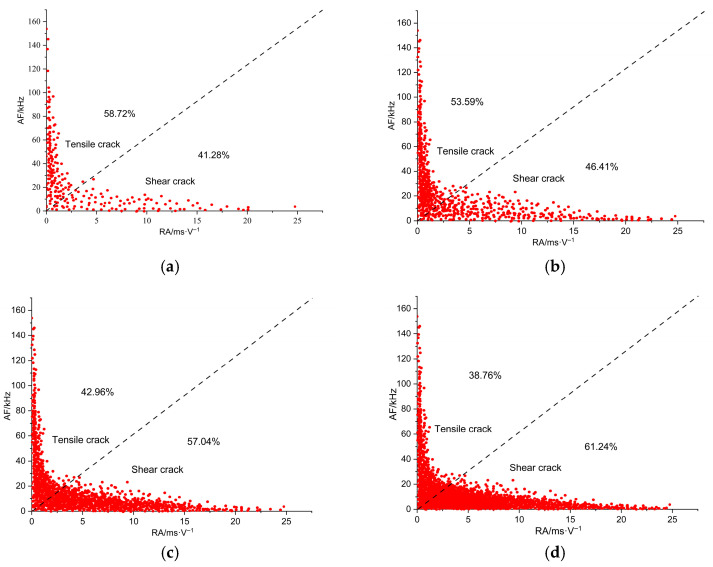
Distribution characteristics of the RA–AF: (**a**) 0~30% peak load; (**b**) 0~50% peak load; (**c**) 0~80% peak load; (**d**) 0~100% peak load.

Concrete damage typically involves two failure modes: shear rupture and tensile rupture. Each mode exhibits distinct characteristics in the RA and AF parameters. Increasing the upper limit of cyclic loading significantly affects the scatter distribution of RA and AF values during the fatigue damage and failure process of anti-slide piles [41]. Throughout the loading cycle, crack propagation manifests differently at various across stages, as illustrated in Figure 10.

During loading from 0% to 30% of the peak load, the AE events associated with tensile rupture are densely clustered, while shear-related events remain relatively sparse. This indicates that tensile failure dominates the early loading stage. When the load reaches 50% of the peak, both tensile and shear events increase significantly. Tensile events show a more concentrated distribution and remain more numerous than shear events, confirming that tensile rupture continues to be the primary failure mode. At 80% of the peak load, event counts for both failure types rise farther, but the increase in shear events surpasses that of tensile events. This shift suggests that shear rupture begins to dominate as the pile approaches failure. Upon reaching peak load, the number of shear events exceeds that of tensile events, demonstrating that the final failure of the pile is governed by shear rupture.

Examining the evolution of damage and failure modes throughout the loading cycle of the anti-slide pile reveals that tensile rupture predominates in the early stage, accompanied by limited shear failure. As loading progresses, both tensile and shear events increase, although tensile failure remains dominant. In the later loading stage, shear rupture events rise sharply and become the prevailing failure mechanism. Combined with the observed failure process of the pile, the abrupt increase in shear events serves as a clear indicator of impending instability and failure of the anti-slide pile [30,42].

### 4.2. The Main Frequency Variation Characteristics of Acoustic Emission During the Damage Evolution Process of Anti-Slide Piles

**Figure 11:**
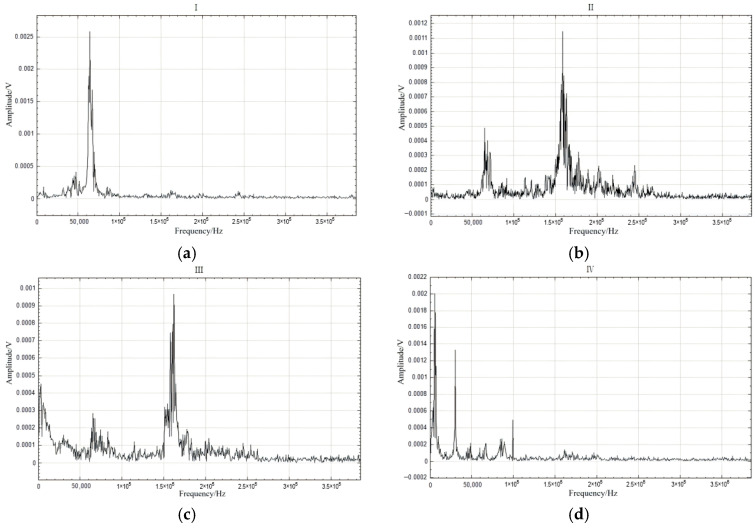
The frequency domain characteristics of signals in each main frequency band. (**a**) Stage I; (**b**) Stage II; (**c**) Stage III; (**d**) Stage IV.

**Figure 12:**
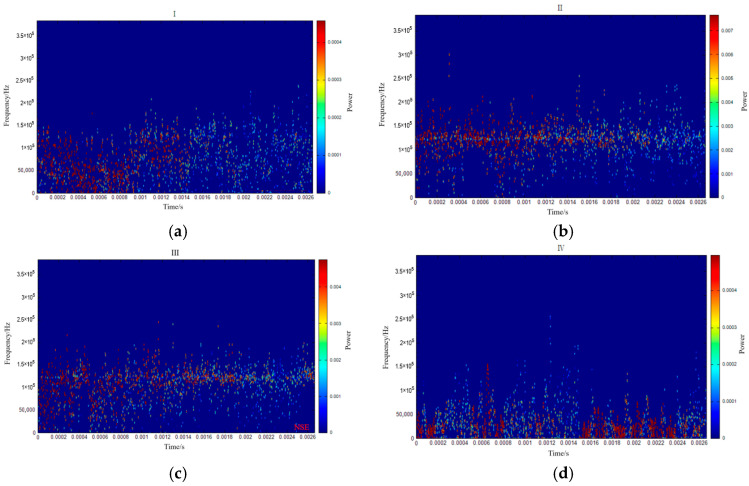
The main time–frequency characteristics of AE signals at each stage. (**a**) Stage I; (**b**) Stage II; (**c**) Stage III; (**d**) Stage IV.

The preceding analysis of the AE signal spectra during the loading of the anti-slide pile classified the evolution of the dominant frequency and its amplitude into four distinct stages, with signals in each stage exhibiting similar characteristics. To identify precursory indicators, a segment of the Hilbert marginal spectrum frequency was extracted for each stage (Figure 11), and the corresponding Hilbert spectrum was analyzed to derive its characteristic features (Figure 12).

Examination of the signal characteristics across the four stages identified three principal frequency bands: 0–50 kHz, 50–125 kHz, and 125–250 kHz, corresponding, respectively, to the low-frequency, medium-frequency, and high-frequency bands. Since the failure of the anti-slide pile occurs in Stage IV, precursors must be extracted from Stages III and IV. The most pronounced distinction between Stages III and IV and Stages I and II is the abrupt rise in low-frequency signals. In the later loading phase approaching failure, the emergence of low-frequency and high-amplitude signals confirms that the accelerated deformation and failure of the anti-slide pile are strongly associated with low-frequency, high-energy AE activity.

However, the abrupt increase in high-energy signals in the low-frequency band alone cannot e directly be considered a reliable precursor to the failure of anti-slide piles. This is primarily because low-frequency signals occur throughout all stages of damage and are not exclusive to the failure phase [43]. As shown in the time–frequency distribution, significant low-frequency components appear in all four stages of loading. Only in Stage IV are low-frequency signals notably more concentrated in terms of quantity or energy. Due to this broad distribution across stages, relying solely on an increase in low-frequency signals makes it difficult to accurately identify whether a retaining structure has reached a critical failure state in practical monitoring.

Therefore, this study further integrates the bending moment response of the anti-slide piles into the analysis. As a direct mechanical indicator of the pile’s loading state, the bending moment is physically linked to the progression of pile damage. By establishing a synergistic relationship between AE signal characteristics and the evolution of the bending moment, the damage development stage can be identified more effectively, thereby enhancing the accuracy and reliability of failure precursor detection in anti-slide piles.

**Figure 13:**
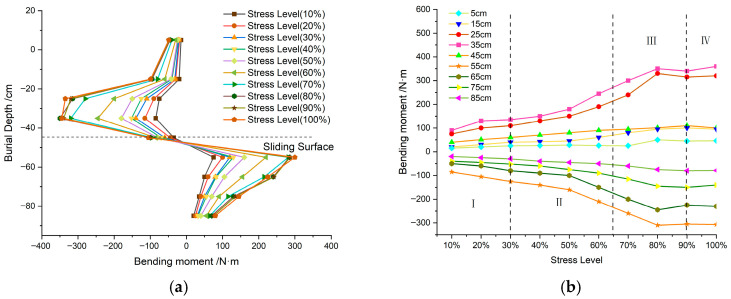
Sliding resistance pile bending moment diagram. (**a**) Bending moment distribution of the anti-slide piles. (**b**) The variation in bending moments at each point of the anti-slide piles.

**Figure 14:**
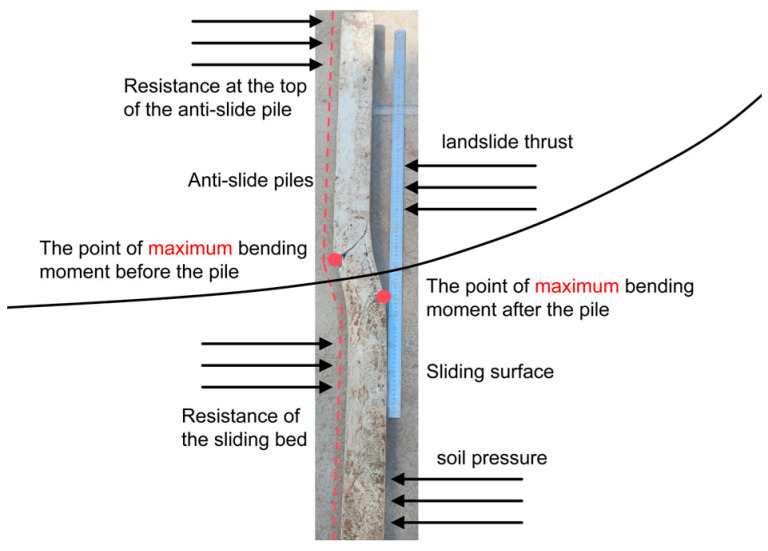
Deformation of the anti-slide piles.

**Figure 15:**
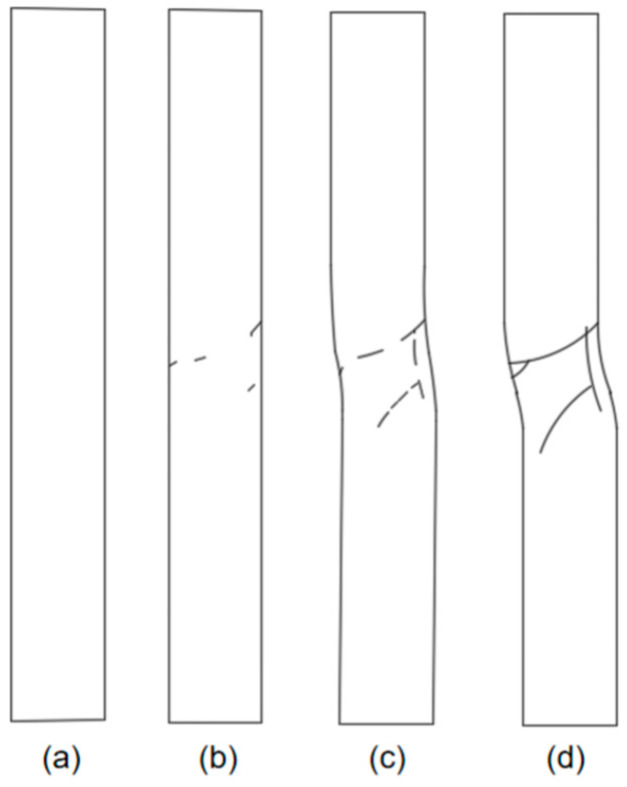
Development of cracks. (**a**) Stage I; (**b**) Stage II; (**c**) Stage III; (**d**) Stage IV.

Based on strain gauge measurements from the reinforcing bars, this study calculated the bending moment distribution in the anti-slide piles during cyclic loading (Figure 13a). The loading process was divided into stages corresponding to 10% increments of the maximum cyclic load, with variations in the bending moment at different pile locations shown in Figure 13b. The soil pressure near the sliding surface is notably concentrated, identifying this zone as critical for the development of pile deformation and damage (Figure 14). The failure process of the anti-slide pile is characterized by the initiation, propagation, and frictional sliding of material cracks, each loading stage exhibiting a distinct evolution mechanism (Figure 15).

Integrated analysis of the AE signal spectra and bending moment response enables a systematic description of the damage evolution in the anti-slide pile across four distinct stages: Stage I (initial compaction, Figure 15a): The dominant AE frequency is centered around 50 kHz. Amplitudes generally remain below 100 mV, with only sporadic increases. Impact counts are limited, and energy release is low. Bending moment changes are slight, with a peak increment of about 50 N·m, corresponding to gradual soil compaction during which the load is not yet fully transferred to the pile. Stage II (load transfer, Figure 15b): The dominant frequency shifts with the load; it moves to 100 kHz between 16 and 28 kN, rises sharply to approximately 150 kHz at 28–36 kN, and then returns to 100 kHz. AE impact counts increase explosively, accounting for roughly 50% of the total, and amplitudes fluctuate strongly, reaching 400–600 mV in some cases. Bending moment enters a period of rapid growth, with a peak increment of 180 N·m; the difference between the moment near the sliding surface and that at the pile ends widens from 130 N·m to 280 N·m, indicating effective action of the landslide thrust. Stage III (damage stabilization and failure transition, Figure 15c): The dominant frequency drops below 100 kHz, and the frequency band narrows. Peak frequencies concentrate in the mid-frequency and high-frequency ranges, amplitudes generally exceed 300 mV, and some medium-amplitude-to-low-amplitude signals disappear. The AE activity is further strengthened, the damage variable rises rapidly to the critical level, the bending moment growth rate slows down, the increasing trend turns to a stable trend, and the pile begins to show macroscopic damage. Stage IV (failure stage, Figure 15d): The dominant frequency continues to weaken to 0–50 kHz, showing an “increase–peak–sharp-decline” pattern. Amplitudes decline significantly, damage variables stabilize, and a large number of low-frequency, high-amplitude signals are released abruptly, along with a sharp rise in energy, ultimately leading to macroscopic instability. Even after through-cracks appear and bearing capacity decreases, AE signals—though weakened—persist, indicating that the pile retains some residual load carrying capacity.

Across these four stages, the AE characteristics and bending moment variations exhibit a clear and coordinated correspondence, providing a reliable foundation for multi-parameter-based damage monitoring and failure warning of anti-slide piles.

This evolution can be interpreted in terms of four consecutive damage phases: Initial damage phase: low-frequency, low-amplitude signals, corresponding to microcrack initiation and elastic deformation. Stable development phase: high-frequency, low-amplitude signals, reflecting intensified micro-fracturing and stable crack propagation. Accelerated development phase: mid-frequency, high-amplitude signals, marking rapid crack growth and macroscopic failure surface formation. Failure phase: low-frequency, high-amplitude signals, indicating complete structural instability. The systematic relationship between AE features and bending moment response at each stage provides a robust basis for AE-based damage monitoring of anti-slide piles.

### 4.3. Recognition of Precursor Information Based on the Energy Ratio of Low-Frequency Band Signals in Acoustic Emission

Previous studies have indicated that low-frequency, high-amplitude signals are closely associated with large-crack propagation during the later loading phase of anti-slide piles and with the concentrated release of accumulated strain energy near failure. To further quantify this relationship, wavelet analysis can be employed to examine the time–frequency distribution of AE signals. This approach enables not only the calculation of energy distribution across different frequency bands, through the energy percentage within each band, but also the characterization of relative changes in low-frequency signal energy across various damage stages, particularly during failure. In doing so, it clarifies the correspondence between low-frequency, high-amplitude AE signals and the energy release patterns accompanying pile failure.

This study employs discrete wavelet transform (DWT) for signal analysis, utilizing the Daubechies wavelet (db4) as the mother wavelet. The db4 wavelet was selected for its excellent time–frequency localization properties and its extensive application and validation in analyzing acoustic emission signals from rock and concrete fractures. Based on a sampling frequency of 796 kHz, the acoustic emission signal was decomposed into eight levels to achieve sufficient frequency resolution for distinguishing the defined frequency bands. The computational window length was set to 1024 samples (approximately 1.29 ms). Threshold selection employed adaptive thresholding based on heuristic principles, with soft thresholding used as the threshold quantization rule. According to the dominant frequency distribution observed in experiments, the acoustic emission signal is divided into three frequency bands: low-frequency band (0–50 kHz), mid-frequency band (50–125 kHz), and high-frequency band (125–250 kHz). The energy in each band is calculated by summing the squares of the corresponding wavelet coefficients at each decomposition level, and the energy proportion of each band is defined as the ratio of that band’s energy to the total signal energy.

**Figure 16:**
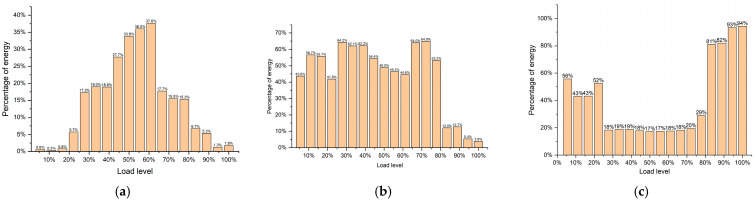
The percentage of energy frequency bands of the AE signals: (**a**) high-frequency; (**b**) medium-frequency; (**c**) low-frequency.

Figure 16 illustrates the evolution of AE signal energy proportions across different frequency bands during the deformation of anti-slide piles. Based the on dominant frequency distributions, the signals are divided into three bands: high-frequency (125–250 kHz), medium-frequency (50–125 kHz), and low-frequency (0–50 kHz).

During the initial compaction stage (load level 0–25%), the high-frequency band contributes very little energy; instead, energy is concentrated in the medium-frequency and low-frequency ranges. At this stage, the soil is compacting, pile deformation remains limited, AE activity is weak, and energy release—consistent with microcrack closure and local stress redistribution—occurs predominantly at medium and low frequencies.

When the load reaches 20–65% (damage development stage), energy shifts toward the medium-frequency and high-frequency bands. As landslide displacement increases and pile deformation expands, microcracks initiate and propagate more rapidly, and medium-frequency to high-frequency signals become dominant [44]. Above 40% load, the proportion of high-frequency energy proportion rises steadily, while the medium-frequency proportion declines, indicating that the pile has entered an accelerated deformation phase and accumulated strain energy is being released. Beyond 65% load (damage stabilization and transition stage), high-frequency energy decreases and medium-frequency energy increases, with the energy peak shifting from high-frequency to medium-frequency. This transition reflects the coalescence of small cracks into larger macroscopic fractures, marking a shift from dispersed micro-fracturing to localized macro-crack development. At 80–90% load (imminent failure stage), low-frequency energy rises sharply to over 80% of the total, while medium-frequency and high-frequency contributions drop markedly. This phase is characterized by abundant low-frequency, high-amplitude signals, corresponding to the rapid loss of pile support and concentrated strain energy release. The abrupt increase in low-frequency energy proportion serves as a key precursor to impending overall slope instability.

In practical monitoring, a pronounced increase in the low-frequency energy proportion of AE signals serves as a direct, quantitative, and sensitive indicator that the landslide–pile system has entered an accelerated deformation and instability critical state. This metric can improve the accuracy and timeliness of real-time landslide early warning systems.

For the early warning criterion, a combined “threshold + duration” approach is employed: an impending failure warning is triggered when the low-frequency energy proportion exceeds 80%, and this condition persists for at least three consecutive computational windows (approximately 3.87 ms). To enhance reliability, auxiliary confirmation conditions are established, requiring that the acoustic emission event rate increases by more than 200% relative to the baseline level, accompanied by a simultaneous decrease in the high-frequency energy proportion to below 10%.

It should be noted that this study currently focuses on analyzing and characterizing the evolutionary patterns of acoustic emission signal spectral features, and the proposed early warning criteria parameters (80% threshold, 3-window duration) are preliminarily determined based on the statistical characteristics of the experimental data. Potential sources of false alarms primarily include transient loading fluctuations and environmental noise interference, which can be effectively suppressed through the aforementioned multi-parameter joint confirmation mechanism and duration constraints. A comprehensive quantitative assessment of false alarm and missed detection rates requires systematic validation experiments with larger sample sizes, which will be a key focus of future research.

### 4.4. Cross-Comparison of the Main Indicators

A comprehensive comparison of the three monitoring indicators reveals that the ring count exhibits the earliest response characteristics. Experimental observations show that the ring count begins to change significantly starting from the 7th loading cycle, with the first noticeable abrupt change occurring at the 12th loading cycle, corresponding to approximately 48% of the peak load. This early response provides the longest warning window, covering about 52% of the time span before final instability. The low-frequency energy ratio exhibits an intermediate response timing. Beyond the 65% load level, the energy share in the low-frequency band (0–50 kHz) begins to rise significantly, surpassing the 80% critical threshold by the 15th loading cycle (approximately 80% load level). Shear event surges (identified via RA–AF analysis) exhibit the latest response: tensile failure events dominate during initial loading (0–30% load), with shear events being rare. Only when the load reaches 80% do shear events begin to outnumber tensile events, marking a fundamental shift in the failure mechanism from tensile-dominated to shear-dominated, as shown in Table 3.

Each of the three indicators has distinct characteristics regarding reliability and implementation complexity. The ringing count provides the earliest warning and is simple to collect, requiring only threshold statistics. However, it is susceptible to environmental noise interference and exhibits a high false alarm rate. The low-frequency energy ratio offers the highest reliability, featuring a clear 80% energy threshold and a well-defined physical correlation with large-scale crack propagation. It also demonstrates strong noise immunity but requires a wavelet transform to calculate the frequency band energy. RA–AF shear event analysis directly reveals the transition process of the failure mechanisms, providing a physical explanation for the evolution of damage patterns from tensile to shear. Although its threshold determination requires manual delineation of the RA–AF boundaries, once the parameters are extracted, its application is straightforward.

Integrating these three indicators creates a layered early warning system that enhances prediction accuracy and addresses the limitations of individual metrics: Ringing counts act as a preliminary screening tool, capitalizing on their early-response advantage. The low-frequency energy ratio employs a clear 80% threshold as its primary criterion. The shear event ratio offers final confirmation of the transition in failure mechanisms preceding instability. Together, these form a comprehensive discrimination chain, progressing from early warning to confirmation of an imminent slip.

## 5. Conclusions

This study conducted a model box test on a landslide–anti-slide pile system to systematically investigate the AE response patterns associated with damage evolution in anti-slide piles under cyclic loading and to establish their correlation with mechanical behavior. The principal conclusions are as follows:

(1) The AE ringing count and energy count of the pile exhibit consistent trends throughout the loading cycle, remaining stable during the early phase and showing a marked change accompanied by a sharp increase in energy release in the later phase. The dominant frequency and amplitude of the AE signals evolve through four distinct stages: low frequency and low amplitude in the initial damage stage, corresponding to microcrack initiation and elastic deformation; high frequency and low amplitude in the stable development stage, reflecting intensified micro-fracturing and new crack propagation; medium frequency and high amplitude in the accelerated development stage, indicating rapid crack growth and macroscopic failure surface formation; and low frequency and high amplitude in the failure stage, signaling complete structural instability. The AE characteristics in each stage display a clear, systematic correlation with the bending moment response, providing a reliable basis for AE-based dynamic damage monitoring of anti-slide piles.

(2) The failure mode of the anti-slide pile evolves characteristically with loading: Tensile failure predominates in the early stage, accompanied by limited shear failure. As loading progresses, shear rupture events increase substantially in the later stage and gradually become dominant. The abrupt rise in shear events can serve as a key precursor to impending pile instability.

(3) A pronounced increase in the proportion of AE signal energy within the low-frequency band directly indicates that the landslide–pile system has entered a critical state of accelerated deformation and impending instability. This surge in low-frequency energy proportion can thus serve as a key precursor indicator of overall slope failure.

Based on the present work and findings, further research may proceed in the following directions, while acknowledging the limitations of the current study:

Due to the limitations of the laboratory setup, differences in scale effects, boundary conditions, and loading rates exist between the indoor model and the complex interactions of a real pile–slope system. These factors may affect the generalizability of the quantitative relationship between damage evolution and AE features. Therefore, future work should include long-term field monitoring at actual landslide sites to systematically validate the applicability and durability of the AE–bending moment integrated approach under varying geological, hydrological, and loading conditions.

Furthermore, AE signals in field environments are susceptible to various noise sources, including ambient vibrations, machinery operation, rainfall, and electromagnetic interference. Although a relatively high signal-to-noise ratio was achieved under the controlled conditions of this study, further development of real-time filtering and feature extraction algorithms tailored to complex background noise is necessary to enhance the method’s robustness and reliability in practical monitoring scenarios.

## Figures and Tables

**Table 1:** Similarity relationship of the model test slides.

**Type**	**Analog Quantity**	Dimension	General Model	Practical Model	This Model
Material properties	Stress σ	${FL}^{-2}$	$K_{\sigma }$	1	20
	Strain ε	-	1	1	1
	Elastic modulus E	${FL}^{-2}$	$K_{\sigma }$	1	20
	Shear modulus $G_{m}$	${FL}^{-2}$	$K_{\sigma }$	1	20
	Compressive strength R	${FL}^{-2}$	$K_{\sigma }$	1	20
	Cohesion C	${FL}^{-2}$	$K_{\sigma }$	1	20
	Friction angle φ	-	1	1	1
	Volumetric weight γ	${FL}^{-3}$	$K_{\gamma }$	1	1
Geometric characteristics	Length L	L	$K_{L}$	$K_{L}$	20
	Linear displacement σ	L	$K_{L}$	$K_{L}$	20
	Angular displacement β	-	1	1	1

**Table 2:** Physical parameters of the test soil.

	Volumetric Weight γ (kN/m^3^)	Moisture Content ρ (γ)	Cohesion C (kPa)	Angle of Internal Friction φ (°)
Landslide body	16.2	16.2	13.2	18.4
Landslide bed	17.8	14.3	15.8	20.4

**Table 3:** Comparison of different monitoring indicators.

Discriminant Indicator	First Significant Response	Corresponding Load	Lead Time for Early Warning
Ring Count Mutation	The 12th loading	~48%	Earliest (52%)
Low-frequency energy ratio > 80%	The 15h loading	~80%	Mid (20%)
Shear cracks > Tensile cracks	The 15th–16th loadings	~80–90%	Latest (10–20%)

## Data Availability

The original contributions presented in this study are included in the article. Further inquiries can be directed to the corresponding author.
